# Contribution of EGFR and ErbB-3 Heterodimerization to the EGFR Mutation-Induced Gefitinib- and Erlotinib-Resistance in Non-Small-Cell Lung Carcinoma Treatments

**DOI:** 10.1371/journal.pone.0128360

**Published:** 2015-05-20

**Authors:** Debby D. Wang, Lichun Ma, Maria P. Wong, Victor H. F. Lee, Hong Yan

**Affiliations:** 1 Department of Electronic Engineering, City University of Hong Kong, Kowloon, Hong Kong; 2 Li Ka Shing Faculty of Medicine, The University of Hong Kong, Pokfulam, Hong Kong; Graduate Institute of Toxicology, TAIWAN

## Abstract

EGFR mutation-induced drug resistance has become a major threat to the treatment of non-small-cell lung carcinoma. Essentially, the resistance mechanism involves modifications of the intracellular signaling pathways. In our work, we separately investigated the EGFR and ErbB-3 heterodimerization, regarded as the origin of intracellular signaling pathways. On one hand, we combined the molecular interaction in EGFR heterodimerization with that between the EGFR tyrosine kinase and its inhibitor. For 168 clinical subjects, we characterized their corresponding EGFR mutations using molecular interactions, with three potential dimerization partners (ErbB-2, IGF-1R and c-Met) of EGFR and two of its small molecule inhibitors (gefitinib and erlotinib). Based on molecular dynamics simulations and structural analysis, we modeled these mutant-partner or mutant-inhibitor interactions using binding free energy and its components. As a consequence, the mutant-partner interactions are amplified for mutants L858R and L858R_T790M, compared to the wild type EGFR. Mutant delL747_P753insS represents the largest difference between the mutant-IGF-1R interaction and the mutant-inhibitor interaction, which explains the shorter progression-free survival of an inhibitor to this mutant type. Besides, feature sets including different energy components were constructed, and efficient regression trees were applied to map these features to the progression-free survival of an inhibitor. On the other hand, we comparably examined the interactions between ErbB-3 and its partners (EGFR mutants, IGF-1R, ErbB-2 and c-Met). Compared to others, c-Met shows a remarkably-strong binding with ErbB-3, implying its significant role in regulating ErbB-3 signaling. Moreover, EGFR mutants corresponding to poor clinical outcomes, such as L858R_T790M, possess lower binding affinities with ErbB-3 than c-Met does. This may promote the communication between ErbB-3 and c-Met in these cancer cells. The analysis verified the important contribution of IGF-1R or c-Met in the drug resistance mechanism developed in lung cancer treatments, which may bring many benefits to specialized therapy design and innovative drug discovery.

## Introduction

**Fig 1:**
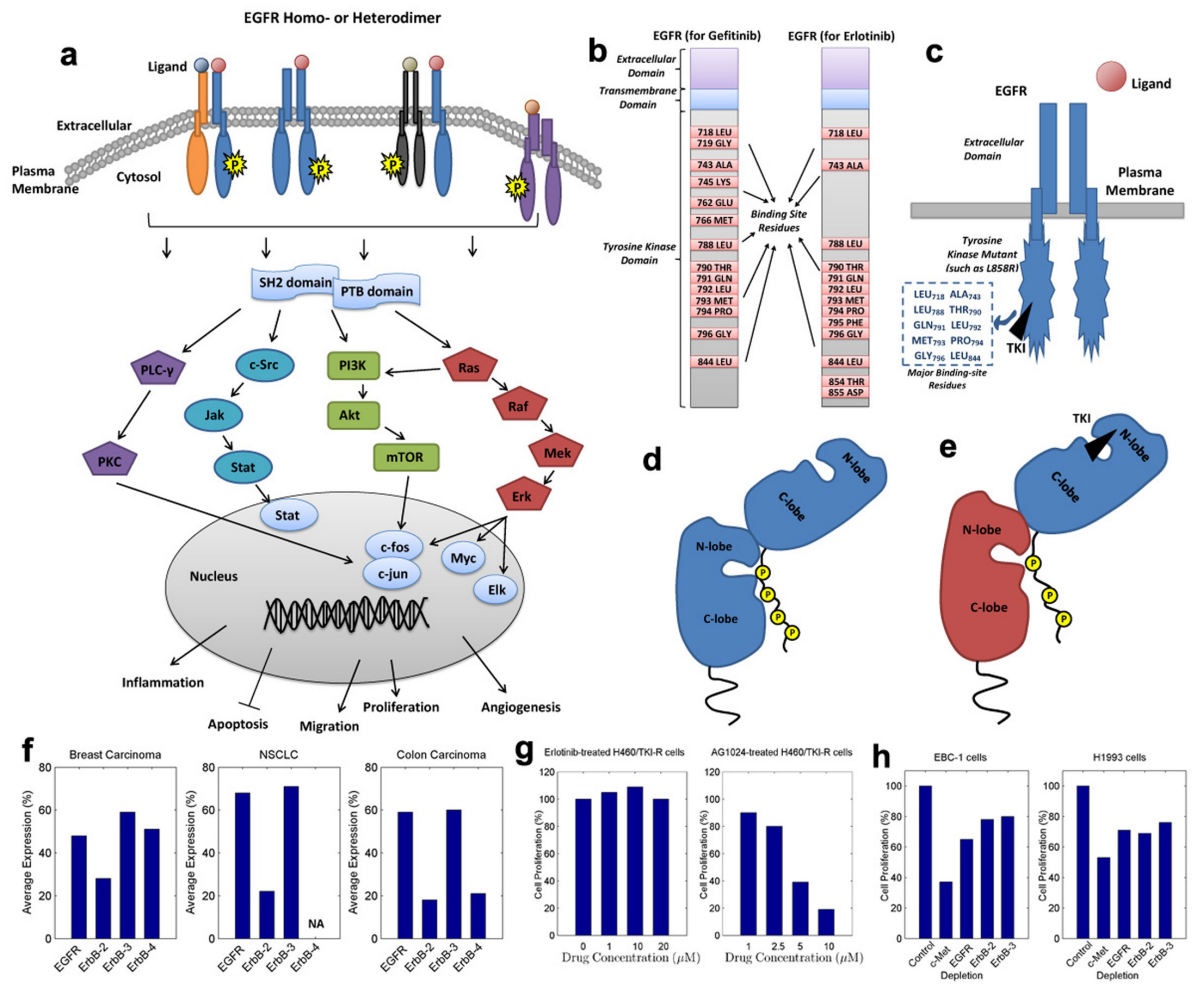
EGFR downstream signaling and EGFR dimerization. (a) A profile of the activation and transduction of EGFR downstream signals. (b) Binding-site residues (of WT EGFR) for two TKIs (Gefitinib and Erlotinib). (c) A sketch of the binding between an EGFR kinase mutant and a TKI, with primary binding-site residues listed. (d) The allosteric mechanism for kinase dimerization of EGFR and its partner. (e) The kinase dimerization for a TKI-blocked EGFR and its potential partner. (f) The average expressions of ErbB receptors in breast carcinoma, NSCLC and Colon carcinoma. (g) The proliferation of H460/TKI-R cells when treated with AG1024 or continuous Erlotinib. (h) The effects of depletion of c-Met, EGFR, ErbB-2 and ErbB-3 on cell proliferation of EBC-1 and H1993 cell lines.

The human epidermal growth factor receptor (EGFR), belonging to the ErbB family of receptor tyrosine kinases (RTK), plays a significant role in the pathogenesis and progression of different carcinoma types, and thus it has become a major topic in cancer research [1–3]. An EGFR-family receptor is composed of an extracellular ligand-binding domain, a hydrophobic transmembrane domain, and an intracellular tyrosine kinase domain [1–4]. A number of ligands, such as EGF, transforming growth factor-*α* (TGF-*α*) and neuregulins (NRGs) [5, 6], can activate EGFR-family receptors, directly provoking them to homo- or heterodimerize [5, 7]. The critical role of dimerization among the EGFR family, in receptor activation and signaling diversity, has long been elucidated [6, 8–10]. Upon dimerization, the catalytic activity of the intracellular tyrosine kinase domain is stimulated, promoting the autophosphorylation of specific tyrosine residues [6, 11]. These phosphorylated residues provide docking sites for downstream signaling molecules (such as Shc, Grb2 and PI3K) containing Src homology 2 (SH2) or phosphotyrosine binding (PTB) domains, the recruitment of which switches on intracellular signals [5, 7]. In EGFR-downstream signaling cascades, *Ras/Raf/Mek/Erk* and *PI3K/Akt/mTOR* are two general pathways [7]. Ultimately, signals will be converted to specific proliferative/apoptotic responses once they arrive the cell nucleus. A profile of this mechanism is displayed in Fig 1a.

Abnormally-amplified signaling can result in malignant cell proliferation (carcinoma) [4, 12]. As the EGFR signaling pathway is well-acknowledged as a leading pathway during the development of many carcinoma types [7, 12, 13], EGFR has become a rational and important therapeutic target [7, 14]. One group of agents that target the kinase domain of EGFR, called tyrosine kinase inhibitors (TKIs), are clinically active and broadly applied in the treatments of cancers such as non-small-cell lung carcinoma (NSCLC) [4, 13, 15]. Such agents bind to the ATP-binding cave of EGFR kinases, leading to the blockade of kinase catalytic activity and to the attenuated signaling pathways. Specifically in NSCLC treatments, two reversible TKIs, gefitinib (*IRESSA*) and erlotinib (*TARCEVA*), are used primarily [16–18]. The rough binding sites (on wild-type EGFR) for gefitinib and erlotinib can be referred to in Fig 1b. More importantly, these TKIs are especially effective in NSCLC patients that harbor activating mutations in their EGFR kinases (Fig 1c) [13, 16]. As reported, mutations clustered around the ATP-binding pocket of EGFR kinase domain frequently occur in NSCLC [15, 16], and different mutation types normally correspond to diverse clinical responses to TKIs [17]. A well-known mutation at the kinase activation loop, the substitution of leucine with arginine at residue site 858 (L858R), showed a good response to these TKIs [4, 15, 19]. However, the efficacy can only last for a short period of time, before drug resistance occurs, probably due to a second mutation such as L858R_T790M at site 790 [15, 20]. One possible reason for this EGFR mutation-driven resistance is the steric interference caused by the mutation [15, 21], which results in a decreased binding affinity between the EGFR mutant and an inhibitor [22]. In fact, it has been verified experimentally that, mutant L858R has a higher binding affinity with Gefitinib or Erlotinib than mutant L858R_T790M [19, 22, 23].

Another major focus in the study of EGFR mutation-induced drug resistance is EGFR dimerization [5–8]. EGFR signaling pathways originate form EGFR dimerization, which produces signaling diversity and is closely associated with cancer development and malignancy [4, 9, 24, 25]. More specially, EGFR heterodimers might have reduced receptor internalization and degradation as compared with homodimers, which thereby can result in enhanced signaling [14]. In this regard, EGFR heterodimers should be a valuable target to be investigated [4, 5]. For the kinase domains of EGFR and its partners, the dimerization mechanism is an allosteric one [20] that highly resembles the one in a CDK/cyclin-like complex [26, 27]. This kinase domain consists of an N-terminal lobe (N-lobe) face, composed mostly of *β*-strands and one *α*-helix, and a C-terminal lobe (C-lobe) face, largely made of *α*-helices. Normally in a dimer, the C-lobe face of one receptor kinase is closer to the N-lobe face coupled with the ATP-binding pocket of the other receptor kinase, encouraging the cross-phosphorylation of the two partners (Fig 1d) [4, 11, 20, 28]. The formation of this asymmetric kinase-dimer has been demonstrated to be an essential prerequisite of receptor activation in the EGFR family [4, 20, 25, 29]. To further investigate the requirements of this activation, Zhang et al. [20] implemented a variety of transfection/cotransfection experiments based a number of EGFR mutants, involving mutations in the ATP-binding site (kinase-dead), N-lobe face and C-lobe face of the dimer interface. Interestingly, according to their outcomes, a kinase-dead receptor with an intact C-lobe face could successfully interact with a partner having an intact N-lobe face and an effective ATP-binding site after ligand stimulation. This study may shed light on our investigation of EGFR mutation-induced drug resistance. Considering the TKI-blocked EGFR kinases as dead ones, they are still able to provide the C-lobe face in the interaction with their dimerization partners as shown in Fig 1e [4, 20], and these interactions are most probably amplified.

Manifestly, other receptors in the EGFR (ErbB) family, namely ErbB-2, ErbB-3 and ErbB-4, are first candidates for EGFR heterodimerization. The EGFR-family members share a high degree of homology in their tyrosine kinase domains, making them favorable dimerization partners [5, 7, 14]. Evidence suggests that cooperation in the ErbB receptor/ligand network can produce signaling diversity and is necessary to induce cell transformation or cancer progression [7, 12]. In Fig 1f, the average expressions of ErbB receptors, reviewed from a series of publications [7, 14, 30–32], are displayed. As shown in this figure, EGFR and ErbB-3 are primarily expressed in human carcinomas (50% ∼ 70%); ErbB-2 is expressed in 20% ∼ 30% of these carcinomas; and the expression of ErbB-4 occurs only in breast and colon carcinomas. In this regard, ErbB-2 and ErbB-3 are considered in our study. ErbB-2 has a powerful tyrosine kinase activity and its extracellular domain is always in an ‘active’ format [2, 33], promoting it to be an ideal partner for all other ErbB receptors [5, 14]. Reciprocally, ErbB-3 is kinase-impaired [6, 14], and thus it is not a preferred partner for a TKI-blocked EGFR. However, it can still be activated by other potential partners such as ErbB-2 [5, 6, 8, 14]. These will be discussed detailedly in the *Results* and *Discussion* sections.

Recently, both *in vitro* and *in vivo* studies have demonstrated the contribution of cross-talk between EGFR and insulin-like growth factor 1 receptor (IGF-1R) to acquired resistance against EGFR-targeted therapies [34–38]. These two receptors interact on multiple levels, either indirectly via common interaction partners, or through a direct association (heterodimer) at the cell surface [34, 39]. A number of clinical studies have shown that treatment of NSCLC cells with EGFR TKIs can induce increased EGFR/IGF-1R heterodimerization and IGF-1R activation [9, 34], enhancing the downstream PI3K/Akt and Mek/Erk pathways [9]. The proliferation of Erlotinib-resistant NSCLC cells (H460/TKI-R) were inhibited when treated with AG1024 (IGF-1R-target), compared with those treated with continuous Erlotinib (EGFR-targeted) (Fig 1g) [9]. It reveals the important role of IGF-1R in recovering signaling pathways during NSCLC progression. More notably, strategies that dually target EGFR and IGF-1R showed better antitumor efficacy than those targeting individual receptors in cancer therapies [40–43]. Accordingly, IGF-1R was investigated as a heterodimerization partner of EGFR in our study.

Amplification of c-Met (MET) [39, 44, 45] has been detected in lung cancer cells developing resistance to Gefitinib or Erlotinib. This c-Met amplification leads to aberrant signaling that is implicated in cell proliferation and migration [46, 47]. Progression of tumors with c-Met amplification are highly dependent on c-Met signaling, mediated by the heterodimers of c-Met with EGFR, ErbB-2 and ErbB-3 [47]. These associations can be markedly reduced by treatment with a c-Met kinase inhibitor or c-Met/ErbB dual inhibitors [47, 48]. As reported in [47], effects of depletion of c-Met, EGFR, ErbB-2 and ErbB-3 on cell proliferation (Fig 1h), of two NSCLC cell lines (EBC-1 and H1993), showed the significant role that c-Met plays in NSCLC progression. Specifically, c-Met can cause drug resistance by the association with ErbB-3 and the posterior activation of PI3K/Akt signaling [46, 47]. In our study, the interactions between c-Met and EGFR/ErbB-3 (especially for ErbB-3) are carefully examined and discussed.

Computational approaches have recently become an efficient and popular alternative for investigating drug resistance mechanisms and innovative drugs [49–52]. In these computational approaches, structure-based methods play an important role [15]. Previously, depending on structure-based approaches, we constructed a personalized drug resistance prediction model for a group of NSCLC patients [15]. These patients all have EGFR kinase mutations, which can be roughly described by the binding affinities between the corresponding EGFR mutants and the TKIs used in the treatments. By combining these simplified EGFR mutation features and the personal features of these patients, an efficient personalized prediction model was constructed, with each patient treated as an input and his/her response level to a specific TKI as an output. However, the characterization of each EGFR mutation may not be sufficient or complete, as the mutation-driven modifications of EGFR-downstream or intracellular signaling pathways are also important. A detailed description of each mutation type can lead to a generalized model for the mapping from a mutation type to a specific TKI resistance level. The progression-free survival (PFS) of an inhibitor, recorded in unit of months or years, is a reliable indicator of the drug resistance level [53]. In brief, screening for diverse EGFR mutation types in lung cancers may identify patients that will response well to different TKIs. In this study we analyzed the interactions between EGFR mutants and their potential dimerization partners (e.g. ErbB-2, IGF-1R and c-Met) coupled with those between ErbB-3 and its partners (e.g. EGFR mutants, ErbB-2, IGF-1R and c-Met). Our study relied on structural templates in the protein data bank (PDB) [54], and adopted the molecular dynamics (MD) simulations as a major analysis technique [55]. Using the enriched EGFR mutation features, exacted from both the interactions with an inhibitor and with a partner, we established a generalized regression model to predict the PFS of an inhibitor to a specific mutant. Besides, we explored the contributions of different factors to the drug resistance level, in these NSCLC treatments. At last, ErbB-3-partner interactions were comparably discussed, to provide a clear outline of ErbB-3 signaling in cancer development.

## Materials and Methods

Approved by the Institutional Review Board of the University of Hong Kong/Hospital Authority Hong Kong West Cluster (HKU/HA HKW IRB), our clinical data were collected from the Queen Mary Hospital. These data were analyzed anonymously, and all the patients involved in the original data collection are no longer alive. Based on these patient data, a series of computational and statistical approaches were implemented for the analysis of drug resistance in NSCLC treatments.

### Data Collection

Specifically, this data set consists of 168 NSCLC patients (stages IIIB and IV) with mutations on exons 18 to 21 of the EGFR tyrosine kinase domain [15]. Before TKI therapies, formalin-fixed paraffin-embedded (FFPE) tumor biopsy samples of these patients were retrieved for EGFR mutation analysis [53, 56]. Overall, they share a total of 37 EGFR mutation types (Tables A to F in S1 File), including residue substitutions, deletions, insertions and double-point mutations. These EGFR mutation types were revealed by direct sequencing [53]. Two TKIs, gefitinib and erlotinib, were the main inhibitors used in the treatments of these patients. We carried out clinical observations on these patients, with the PFSs of their corresponding inhibitors recorded (in unit of months).

### MD Simulations

Initially, we determined the 3D structure of each EGFR kinase mutant based on homology modeling, using *scap* [57] and *loopy* [58]. *scap* is a program for side-chain prediction and residue mutation. It provides a series of side-chain rotamer libraries [59] for side-chain packing. Based on a structural template, *scap* predicts protein residues according to rotamer selection, steric feasibility and energy preference [57, 60]. Mutations such as point mutations were handled by *scap* in our study. In this modeling, the AMBER force field was selected in accordance with following AMBER simulations, and the large side-chain rotamer library was used for a thorough structural search. Similarly, *loopy* predicts loops and mutated sequences for protein chains. Multiple initial conformations of the predicted/mutated sites are modeled using *ab-initio* methods [58, 60], and each conformation is then closed by a random tweak method [61]. Side-chain assessment [57], steric feasibility and energy preference (colony energy) [58] are combined to make the best prediction. Specifically, deletion- and insertion-mutations were modeled by *loopy*, and the AMBER force field was applied as well. For each sampled structure we executed a rough 5000-step AMBER minimization [55] for structural refinement. The quantum mechanics/molecular mechanics (QM/MM) mechanism was implemented in this refinement, with the mutated neighborhood regarded as a QM region and the rest as a MM region.

Once we obtained the refined structures of these mutants, we implemented two groups of experiments using the AMBER software suite [55]. Specifically, the experiments focused on analyzing the protein-ligand interactions and protein-protein interactions that concern our modeled mutants. The AMBER software suite was designed for biomolecular simulations and analysis, normally with a force field specified. Eq 1 shows a simple additive force field, adopted by AMBER.

$$\begin{array}{ccc} V(r) & = & E_{bonded}+E_{nonbonded}\\ & = & \sum_{bonds}K_{b}{\left(b-b_{0}\right)}^{2}+\sum_{angles}K_{\theta }{\left(\theta -\theta_{0}\right)}^{2}\\ & + & \sum_{dihedrals}\left(V_{n}/2\right)\left(1+cos\left[n\phi -\delta \right]\right)\\ & + & \sum_{nonbij}\left(A_{ij}/r_{ij}^{12}\right)-\left(B_{ij}/r_{ij}^{6}\right)+\left(q_{i}q_{j}/r_{ij}\right) \end{array}$$ (1)

In our implementations, the ff99SB force field was selected for proteins, due to its careful management of the helical and extended regions of protein backbones. Small ligands were handled by the gaff force field. Prior to any simulations, a solvent environment should be generated. A truncated octahedron water box, based on the common TIP3P water model, was created for each solute with a 10-angstrom ($\overset{{}^{\circ}}{A}$) buffer in each direction. The 10-$\overset{{}^{\circ}}{A}$ buffer was selected to compensate the large computational costs.

At the early stage, we studied the mutant-inhibitor systems [15]. Each mutant structure was aligned to complex 2ITY [19] or 1M17 [62], assisted by the UCSF chimera [63], to construct the mutant-gefitinib or mutant-erlotinib system. Consecutively, we executed a minimization, a heating, a density equilibration, a constant pressure equilibration and a production MD simulation, in the aforementioned solvent environment. On our computer sever with 12 3.47-GHz processors, the series of operations approximately took 18 hours. We also tested the simulations on a High Performance Computer Cluster (HPCC) [64], composed of a master node and 44 computer nodes with each having 16 cores. A 5-node computation for each system can reduce the computational time to less than 3 hours.

On the other hand, we investigated the mutant-partner systems. The asymmetric dimer structure proposed in [20] was regarded as our dimer template. A potential dimerization partner of EGFR, namely ErbB-2 or IGF-1R, was aligned to the position in the template where its N-lobe face participates in the interactions with the C-lobe face of an EGFR mutant. c-Met has a larger structural difference from ErbB-2 or IGF-1R in the N-lobe face, and the dimer template may not be suitable for it to provide the N-lobe face, therefore we align it to the position where its C-lobe face interacts with EGFR, as a supplementary study. ErbB-3 has an impaired kinase domain and acts as an ideal co-receptor, thus in our study it provides the C-lobe face in the communications with its potential partners. Posterior to the computational solvation of each acquired dimer complex, the dynamics of the system were simulated.

The entire system should be equilibrated prior to the crucial MD simulation. We employed a similar series of equilibrating operations as for the mutant-inhibitor systems, including

- a short 1000-step minimization (the first half with the steepest descent steps) with weak position restraints (weight of 2.0) on the dimer to remove bad contacts,
- a following 1000-step minimization (the first half with the steepest descent steps) on the whole system without restraints,
- a 25-ps heating from 100 to 300 Kelvin (K) with week restraints on the dimer,
- a 25-ps density equilibration with weak restraints on the dimer,
- a 250-ps constant pressure equilibration at 300 K.

Owing to the higher complexity of a protein-protein system, compared to a protein-ligand system, we implemented two minimizations on the system with and without restraints on the dimer. Besides, the SHAKE setting and the temperature control were the same as those in [15], and the time step was confined to 1 femtosecond (fs) to adjust the settings. The equilibration of each system was verified through its temperature, density, energy and the backbone RMSD curve of the dimer complex.

Consecutively, we generated the production MD simulation at an interval of 2 fs for 2 ns, where we collected trajectory frames at a step of 10 ps and 200 frames in each trajectory. Importantly, the stabilization of each system was confirmed by the backbone RMSD curve of its mutant-partner complex, to guarantee a reliable calculation of the binding free energy. Through applying the previously-mentioned 5-node computer cluster, the total computational cost for each system can be reduced to less than 9 hours.

### Binding Free Energy Calculation Based on the MM/GBSA model

The production MD simulations produced the motion trajectories of each solvated system, and the binding free energy of the involved mutant-partner complex was calculated based on these trajectories. Binding free energy is a reliable estimate of the binding affinity of a solvated receptor-ligand system (protein-ligand or protein-protein). The fundamental theory is the thermodynamic cycle, characterized by the following equation.

$$\begin{array}{c} \Delta G_{Bind,Solv}=\Delta G_{Bind,Vacuum}+\Delta \Delta G_{Solv} \end{array}$$ (2)

Here Δ*G*_*Bind*,*Solv*_ or Δ*G*_*Bind*,*Vacuum*_ represents the free energy difference between the bound and unbound states of a complex, in a solvent or vacuum environment. ΔΔ*G*_*Solv*_ is the difference between the solvation free energy of the complex and that of the two binding partners (Eq 3).

$$\begin{array}{c} \Delta \Delta G_{Solv}=\Delta G_{Solv,Complex}-\Delta G_{Solv,Receptor}-\Delta G_{Solv,Ligand} \end{array}$$ (3)

The Molecular Mechanics/Generalized Born Surface Area (MM/GBSA) calculations performed by AMBER [55] can efficiently estimate these free energy differences. Δ*G*_*Bind*,*Vacuum*_ is approximated by the averaged interaction energy between the two binding units, with the entropy component considered. The solvation free energy Δ*G*_*Solv*_ is typically decomposed into the electrostatic and non-electrostatic components in the following equation,

$$\begin{array}{c} \Delta G_{Solv}=\Delta G_{el}+\Delta G_{nonel} \end{array}$$ (4)

Two types of interactions contribute to Δ*G*_*nonel*_, namely the favorable Van der Waals attraction between the solute and the water molecules, and the unfavorable cost of breaking the structure of the water around the solute. Δ*G*_*el*_ is estimated as follows,

$$\begin{array}{c} \Delta G_{el}=-\frac{1}{2}\sum_{ij}\frac{q_{i}q_{j}}{f_{GB}\left(r_{ij},R_{i},R_{j}\right)}\left(1-\frac{exp[-Kf_{GB}]}{\varepsilon }\right) \end{array}$$ (5)

where *q* and *R* are the charge and radius respectively of an atom, *r* indicates the distance between two atoms, *ɛ* is the solvent dielectric, *f*_*GB*_ and *K* are controllable parameters. The energy form can be further simplified into Eq 6, with *ρ*_*i*_ standing for the van der Waals radius of an atom.

$$\begin{array}{c} \Delta G_{el}=-\frac{q_{i}^{2}}{2\rho_{i}}\left(1-\frac{1}{\varepsilon }\right) \end{array}$$ (6)

Specifically, the binding free energy Δ*G*_*Bind*,*Solv*_ is comprised of succeeding components, Van der Waals forces (VDW), electrostatic interactions (EEL), and the polar (EGB) and non-polar (ESURF) contributions of the solvation free energies. In order to provide an efficient computation, AMBER normally neglects the entropy contribution to these energies for similar systems. For the WT protein and each EGFR mutant, we calculated their binding free energies with gefitinib, erlotinib, ErbB-2, ErbB-3, IGF-1R and c-Met, respectively. The distributions of these energies and their components were comparably and specifically analyzed.

As discussed above, ErbB-3 is a kinase-impaired co-receptor, suggesting it may not be as a favorable partner as ErbB-2, IGF-1R or c-Met, for TKI-blocked EGFRs. However, it can be a potential partner for ErbB-2, IGF-1R or c-Met. In this regard, we considered the EGFR-partner interactions and the ErbB-3-partner interactions separately, leaving the ErbB-3-related study as a supplementary one. First, we explored the dependence between the EGFR-partner/EGFR-inhibitor interaction patterns and the PFSs of our NSCLC patients, after which we compared and discussed different ErbB-3-partner interactions.

### Regression to the PFS Based on Binding Free Energy Components of EGFR Mutant-partner/mutant-inhibitor Systems

For each patient, the PFS of the inhibitor used in his or her therapy was recorded. All these patients were grouped according to their EGFR mutation types and the involved inhibitors (gefitinib and erlotinib). Each pair of a mutation type and an inhibitor corresponds to a PFS, which is the median or average of the PFSs regarding the group of patients harboring this mutation and treated with this inhibitor. When calculating the average, both the largest and lowest values were discarded to reduce possible bias. Now we focus on a generalized investigation on the resistance level of a specific mutation type to an inhibitor, for the scenario where patient data are deficient. This differs from the personalized study concerning each patient [15].

Posterior to an outlier-removal operation on these PFS values, we simply adopted the regression trees [65] for mapping the aforementioned energies or energy components to these PFS values. The classification and regression trees (CART) are a simple but efficient method for handling classification and progression problems. Given a set of samples, the mechanism starts at a single node, and partitions the node in a binary way when the largest impurity reduction is achieved. The partitioning continues until the error reaches a stopping criterion. Common impurity measures include entropy (Eq 7) and Gini-index (Eq 8),

$$\begin{array}{c} E\left(X\right)=-\sum_{x\in X}p\left(x\right)log\;p\left(x\right) \end{array}$$ (7)

$$\begin{array}{c} G\left(X\right)=1-\sum_{x\in X}p{\left(x\right)}^{2} \end{array}$$ (8)

where **x** indicates a possible value of the variable **X**, and *p*(**x**) represents the frequency of **x** in a discrete case.

Two common indexes, the relative absolute error (RAE) and root relative square error (RRSE), were selected to evaluate the regression performances in our implementations. These two measures can be formulated as Eqs 9 and 10,

$$\begin{array}{c} RAE=\frac{\sum_{i=1}^{n}\left|P_{i}-T_{i}\right|}{\sum_{i=1}^{n}\left|T_{i}-\frac{1}{n}\sum_{i=1}^{n}T_{i}\right|} \end{array}$$ (9)

$$\begin{array}{c} RRSE=\sqrt{\frac{\sum_{i=1}^{n}{\left(P_{i}-T_{i}\right)}^{2}}{\sum_{i=1}^{n}{\left(T_{i}-\frac{1}{n}\sum_{i=1}^{n}T_{i}\right)}^{2}}} \end{array}$$ (10)

where *P*_*i*_ and *T*_*i*_ represent the predicted and target values of the *i*th sample, respectively. Normally, the RAE or RRSE values fall into the range of [0, 1], and a favorable performance corresponds to a value approximating to zero. Based on different sets of energy features, including the total binding free energies or energy components of the EGFR mutant-inhibitor/mutant-partner systems, we performed the regression analysis and evaluated the results using the RAE and RRSE values.

### Investigation of ErbB-3-partner Interactions

Ultimately, interactions between ErbB-3 and its partners, characterized similarly using binding free energy and its components, were analyzed. All the patient-derived EGFR mutants coupled with c-Met, ErbB-2 and IGF-1R, were considered in our analysis as an ErbB-3 partner. The interaction patterns of these ErbB-3-partner systems were comparably examined, especially for those involving well-know EGFR mutants (L858R, L858R_T790M) and c-Met.

## Results and Discussion

### Three-dimensional Templates

**Fig 2:**
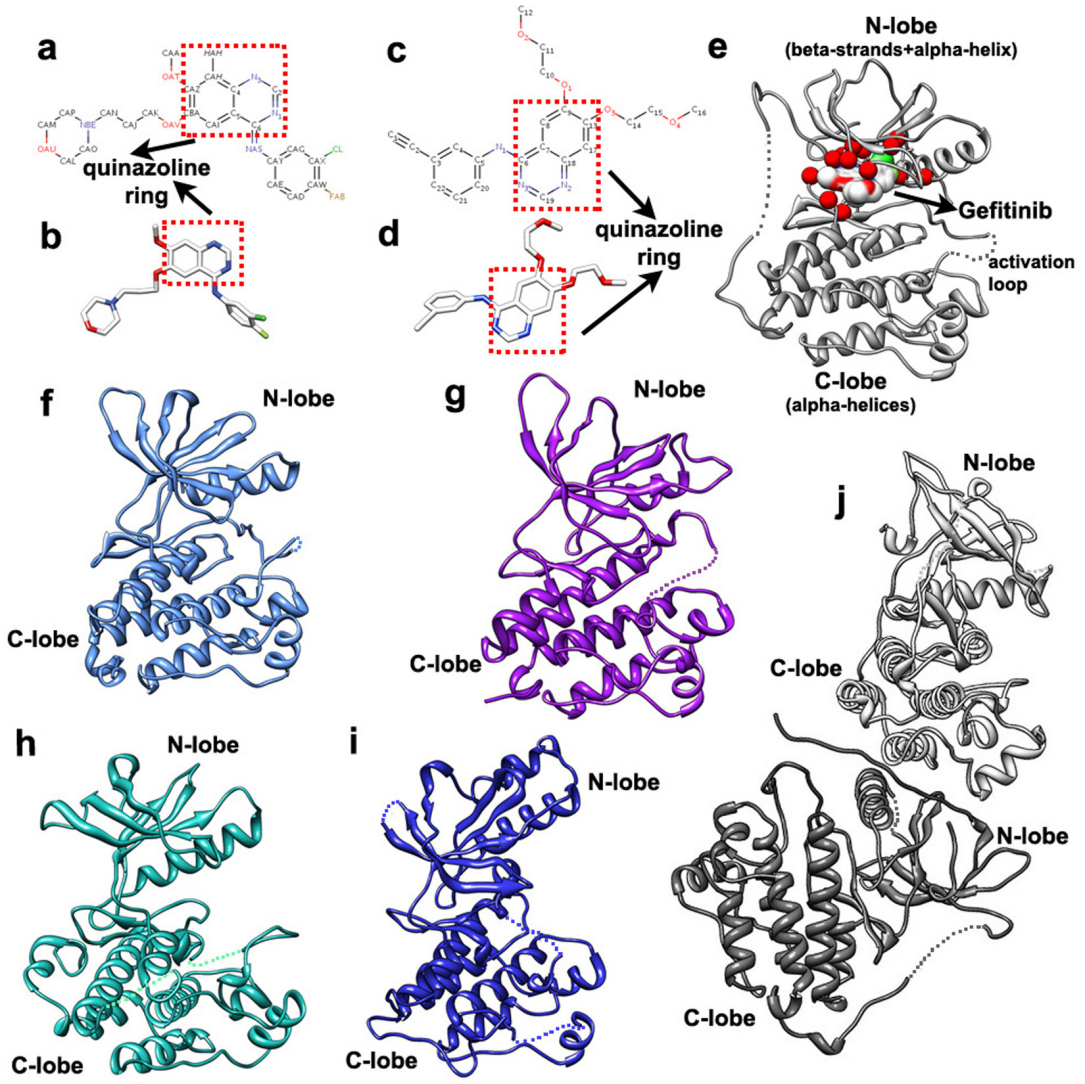
The three-dimensional templates in our structural analysis. (a) and (b), The structures of gefitinib (PDB: 2ITY), with its atoms and essential pharmacophore (quinazoline ring) shown. (c) and (d), The structures of erlotinib (PDB: 1M17), with its atoms and essential pharmacophore (quinazoline ring) shown. (e) Complex 2ITY (in PDB), composed of an active WT EGFR kinase (N-lobe + C-lobe) and an inhibitor (gefitinib); major gefitinib-interacting residues are labeled as red balls. (f) to (i), The structures of the kinase domains of ErbB-2 (PDB: 3PP0), ErbB-3 (PDB: 4RIW), IGF-1R (PDB: 1K3A) and c-Met (PDB: 1R1W), respectively. (j) The asymmetric dimer that is formed by two WT EGFR kinases.

Two inhibitors, gefitinib and erlotinib, are broadly applied in the NSCLC treatments [66, 67]. Specifically for our 168 patients with stages IIIB and IV NSCLC, 137 of them were treated with gefitinib and the rest (31 cases) with erlotinib [15, 53]. The 3D structures of these two inhibitors, respectively separated from complexes 2ITY [19] and 1M17 [62] in the PDB [54], are exhibited in Fig 2b and 2d respectively, and these structures were utilized as ligand templates in our major MD simulations. Component atoms of gefitinib and erlotinib are shown in Fig 2a and 2c, with their essential pharmacophore (quinazoline ring) highlighted in a red box.

Besides, the patients possess 37 types of EGFR mutations (Tables A to F in S1 File), which correspond to mutations in exons 18 to 21 of the EGFR tyrosine kinase domain. To gain a deeper insight into these mutations, we firstly decoded their 3D structures based on the corresponding protein sequences and a 3D template, and these predicted mutants were used in our succeeding MD simulations. Two WT EGFR kinase proteins (active form) in complexes 2ITY and 2ITZ [19] were regarded as our templates for the mutant prediction. Complex 2ITY is displayed in Fig 2e, where the N-lobe, C-lobe, and primary drug-interacting residues of the kinase domain are shown. Majority of mutants were modeled based on 2ITY, which contains the wild type (WT) EGFR kinase. Those L858R-related mutants, such as L858R_T790M, were handled using 2ITZ that involves the kinase domain of mutant L858R. Both 2ITY and 2ITZ were resolved in [19], and thus they share similar structural details and can be comparably used as our templates. At the early stage, we examined the binding affinity of each mutant-inhibitor system [15], which was an important index for studying the potency of an inhibitor (or drug resistance level) to a specific EGFR kinase mutant. Complex 2ITY was adopted as a mutant-inhibitor template for a rough formation of the system, prior to the AMBER minimizations or simulations.

The regulation of the EGFR downstream signaling pathways is vital for the efficacy of an inhibitor or the occurrence of drug resistance, thus analyzing the EGFR-partner interactions in these pathways can provide valuable clues to our study. The homo- or hetero-dimerization of EGFR with its partners is the origin of these signaling pathways, and it is quite an important factor for the downstream signaling regulation. Based on our early study on the mutant-inhibitor systems, we extended our investigation to that on the mutant-partner systems. Potential dimerization partners of EGFR, namely ErbB-2 (PDB: 3PP0) [68], IGF-1R (PDB: 1K3A) [69] and c-Met (PDB: 1R1W) [70], were analyzed in our work. Since ErbB-3 (PDB: 4RIW) [28] is an efficient co-receptor, ErbB-3-partner interactions were considered as well. The 3D structures of these proteins are shown in Fig 2f–2i. At last, the asymmetric dimer (Fig 2j) [20] was employed as a dimer template for EGFR mutant-partner and ErbB-3-partner systems, to provide a rough input to the primary MD simulations. For simplicity, we will abbreviate ‘EGFR mutant’ to ‘mutant’ in the following sections.

### System Construction and MD Simulation Results

**Fig 3:**
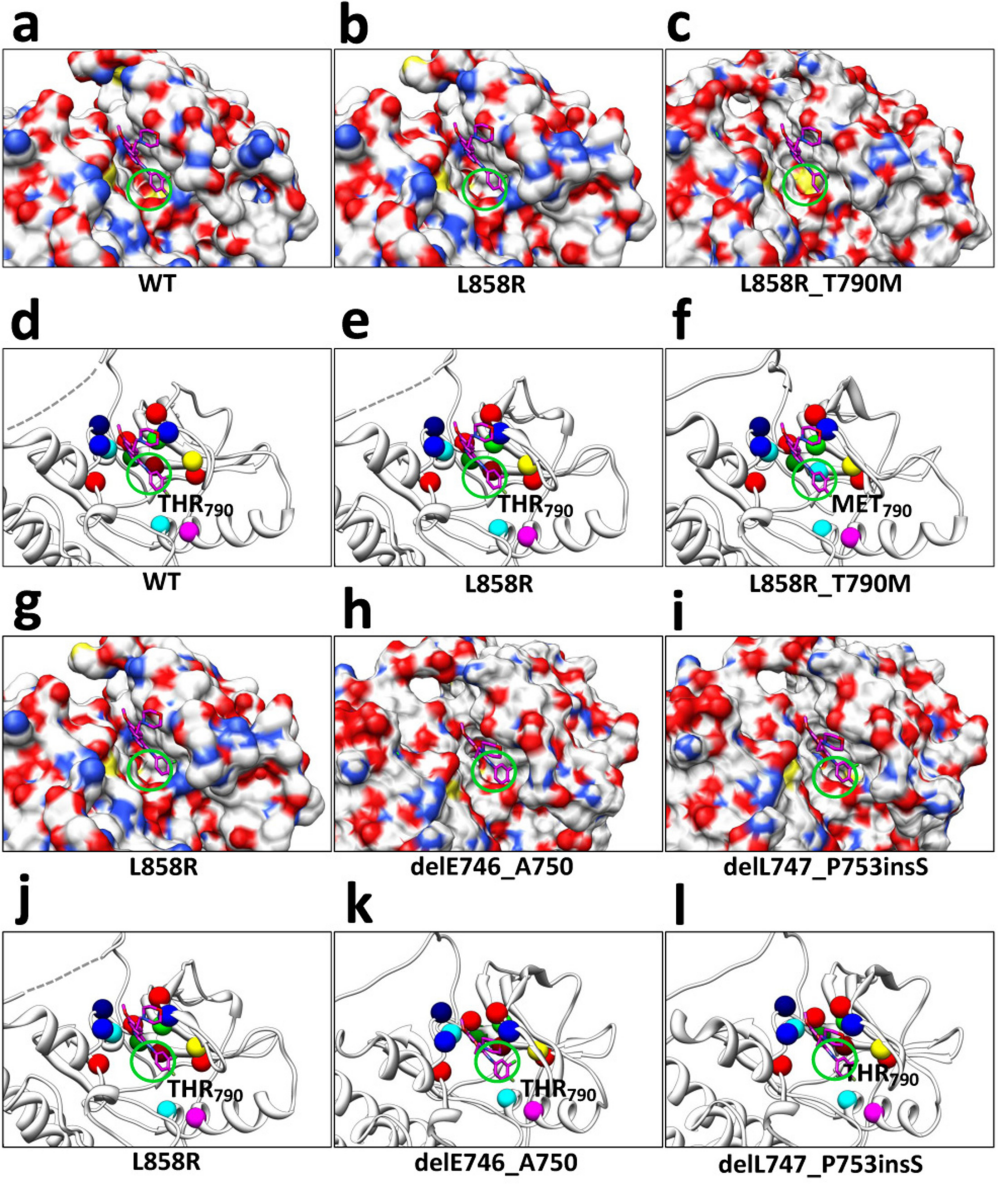
Structural comparison of the TKI-binding caves of several EGFR WT or mutant proteins, with Gefitinib exhibited. (a) to (c), Structural comparison among TKI-binding caves of WT EGFR, mutant L858R, and mutant L858R_T790M; major differences at the gatekeeper position (*790*) are labeled with green circles. (d) to (f), Binding-site residues of WT EGFR, mutant L858R, and mutant L858R_T790M, with residues colored as follows, LEU (red), ALA (green), GLY (blue), LYS (yellow), GLU (magenta), MET (cyan), THR (dark red), GLN (dark green), and PRO (dark blue). (g) to (i), Respective TKI-binding caves of mutants L858R, delE746_A750 and delL747_P753inS, with major structural differences at the gatekeeper position labeled. (j) to (l), Colored binding-site residues of mutants L858R, delE746_A750 and delL747_P753inS.

As described previously, the 3D structures of the 37 EGFR mutants were translated from the protein sequences based on a template. *Scap* [57] and *loopy* [58] handled the structure prediction. Specifically, *scap* deals with side chain substitutions (point mutations), which are notated by the abbreviations of the amino acid residues at the mutated positions, such as L858R. In addition, *loopy* manages residue deletions and insertions, which respectively are notated by prefixes ‘del’ and ‘ins’, such as delE746_A750 and delL747_A755insSKG. Also, duplications of residue, regarded as a sort of residue insertion, are notated using prefix ‘dul’ such as dulH773. Besides, a double-point mutation is named by a connection of two single-point mutations, such as L858R_T790M. Some detailed modeling results can be found in our early studies [15]. Since the mutations normally occur at the TKI-binding site or its neighborhood of EGFR tyrosine kinase domain, we further conducted a structural investigation on the TKI-binding caves of our mutants. Fig 3 shows several instances, including WT EGFR (Fig 3a), L858R (Fig 3b and 3g), L858R_T790M (Fig 3c), delE746_A750 (Fig 3h) and delL747_P753insS (Fig 3i). For simplicity, we merely observed the gatekeeper position (*790*), which was regarded as an important sign for drug efficacy or resistance in early studies [22]. Fig 3a–3c present a comparison among the WT EGFR, mutant L858R, and mutant L858R_T790M. L858R is a well-know good responder to TKIs, and its flat gatekeeper position shows a larger affinity with Gefitinib (Fig 3b). WT EGFR (Fig 3a) and L858R_T790M (Fig 3c) contain a more concave or convex gatekeeper position, which may resist the binding with TKIs. To further clarify the structural difference, we present the major binding-site residues in Fig 3d–3f, with the gatekeeper residues labeled. Moreover, the comparison among L858R, delE746_A750, and delL747_P753insS are shown in Fig 3g–3l. This implies, compared to delL747_P753insS, L858R or delE746_A750 may have a better affinity with TKIs.

Before the major MD simulations, each mutant structure was roughly minimized for 5000 AMBER steps. In our major AMBER simulations, we implemented two groups of experiments. One focused on the mutant-inhibitor systems that each involves an EGFR mutant and an inhibitor (gefitinib or erlotinib), and the other concerned with the mutant-partner (or ErbB-3-partner) systems with each comprising a mutant and a dimerization partner (ErbB-2, IGF-1R or c-Met). For the first group, each mutant structure was aligned to the template complexes to form its mutant-inhibitor complexes. In the AMBER minimizations or simulations, the generalized AMBER force field (gaff) and the AM1-BCC charge method [71] were employed to handle the inhibitors, prior to the computational solvation of each system into a water environment. After a series of equilibrating operations, consisting of a short minimization (1000 steps), a heating phase (50 picoseconds (ps)), a density equilibration (50 ps) and a constant pressure equilibration (500 ps), we carried out the crucial MD simulation for 2 nanoseconds (ns). The stabilization of each system was verified by the backbone root-mean-square deviation (RMSD) curve of the corresponding mutant in the production MD simulation phase (2 ns) [15].

**Fig 4:**
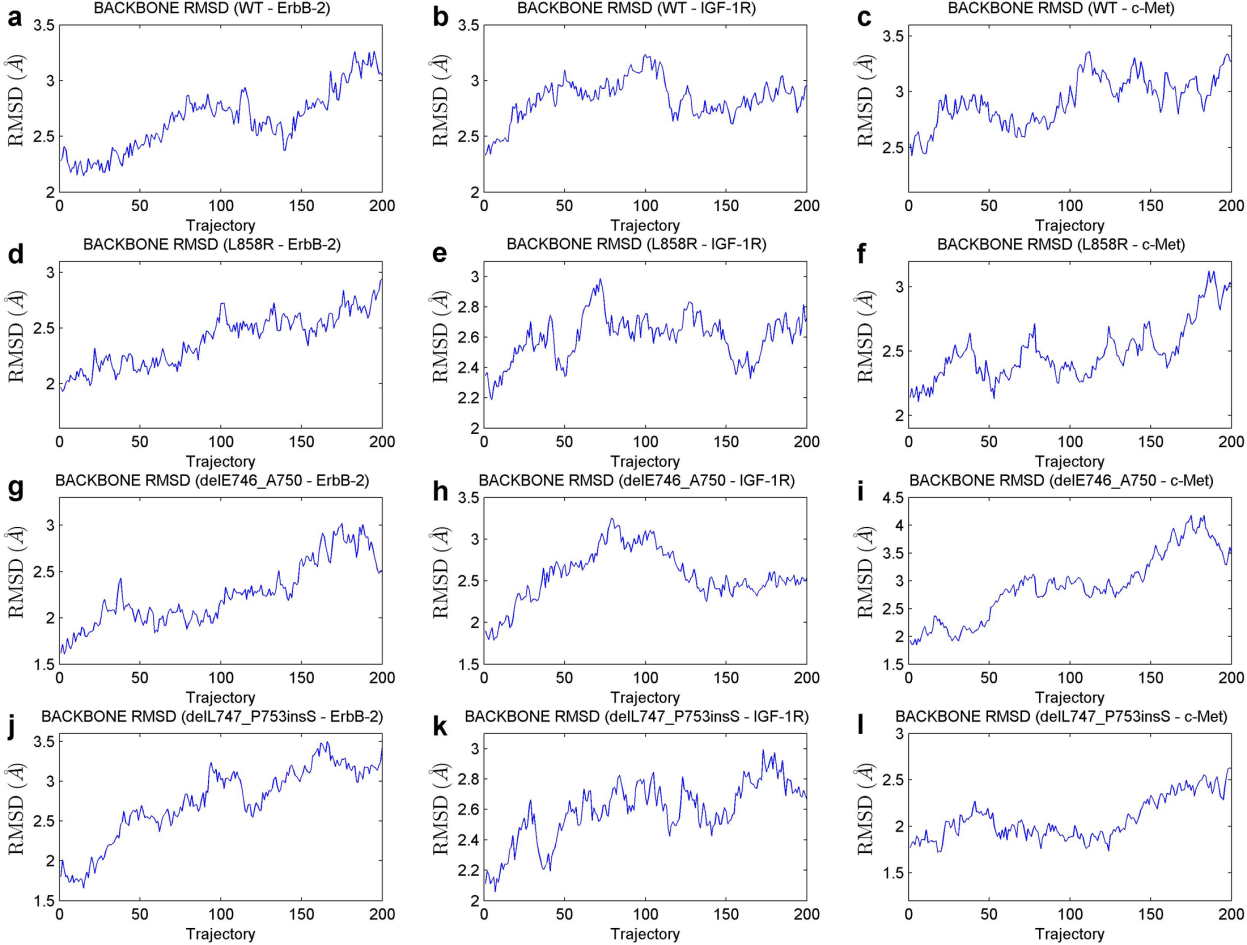
Backbone RMSD curves of several EGFR mutant-partner systems in the 2-ns production MD simulations. (a) to (c), The RMSD curves for the WT—ErbB-2, WT—IGF-1R and WT—c-Met systems, respectively. (d) to (f), The curves for the mutant L858R-related systems. (g) to (i), The scenarios where the mutant delE746_A750 is involved. (j) to (l), The curves for the systems concerning mutant delL747_P753insS.

Aside from the analysis on the mutant-inhibitor systems, we carried out a series of simulations on the newly-constructed mutant-partner or ErbB-3-partner systems. After computationally solvating each system into a water environment, we equilibrated the system at first, similar to the aforementioned operations for the mutant-inhibitor systems. These equilibrating steps encompass two short minimizations with and without restraints (each for 1000steps), a heating phase (25 ps), a density equilibration (25 ps) and a constant pressure equilibration (250 ps). Afterwards, a production MD simulation of 2 ns was implemented. The stabilizations of these systems were verified as well, and Fig 4 shows the RMSD curves of several important EGFR mutant-partner complexes, indicating acceptable stabilizations of their corresponding systems.

### Characterization of Molecular Binding Affinity

Based on the production MD simulation trajectories, we characterized the binding affinity of a solvated mutant-inhibitor or mutant-partner/ErbB-3-partner system. Binding free energy is a favorable estimate of this binding affinity, which can be derived by performing Molecular Mechanics / Generalized Born Surface Area (MM/GBSA) calculations [55] using AMBER. The GB model efficiently computes different types of free energy differences, and further constructs the binding free energy components of VDW, EEL, EGB and ESURF. We calculated the binding free energy and its components for each system (Tables A to F in S1 File), and regarded those concerning the WT protein (WT—Gefitinib, WT—Erlotinib, WT—ErbB-2, WT—IGF-1R and WT—c-Met systems) as baselines for normalization of mutant-inhibitor/mutant-partner interactions. ErbB-3-partner interactions will be discussed separately afterwards.

**Fig 5:**
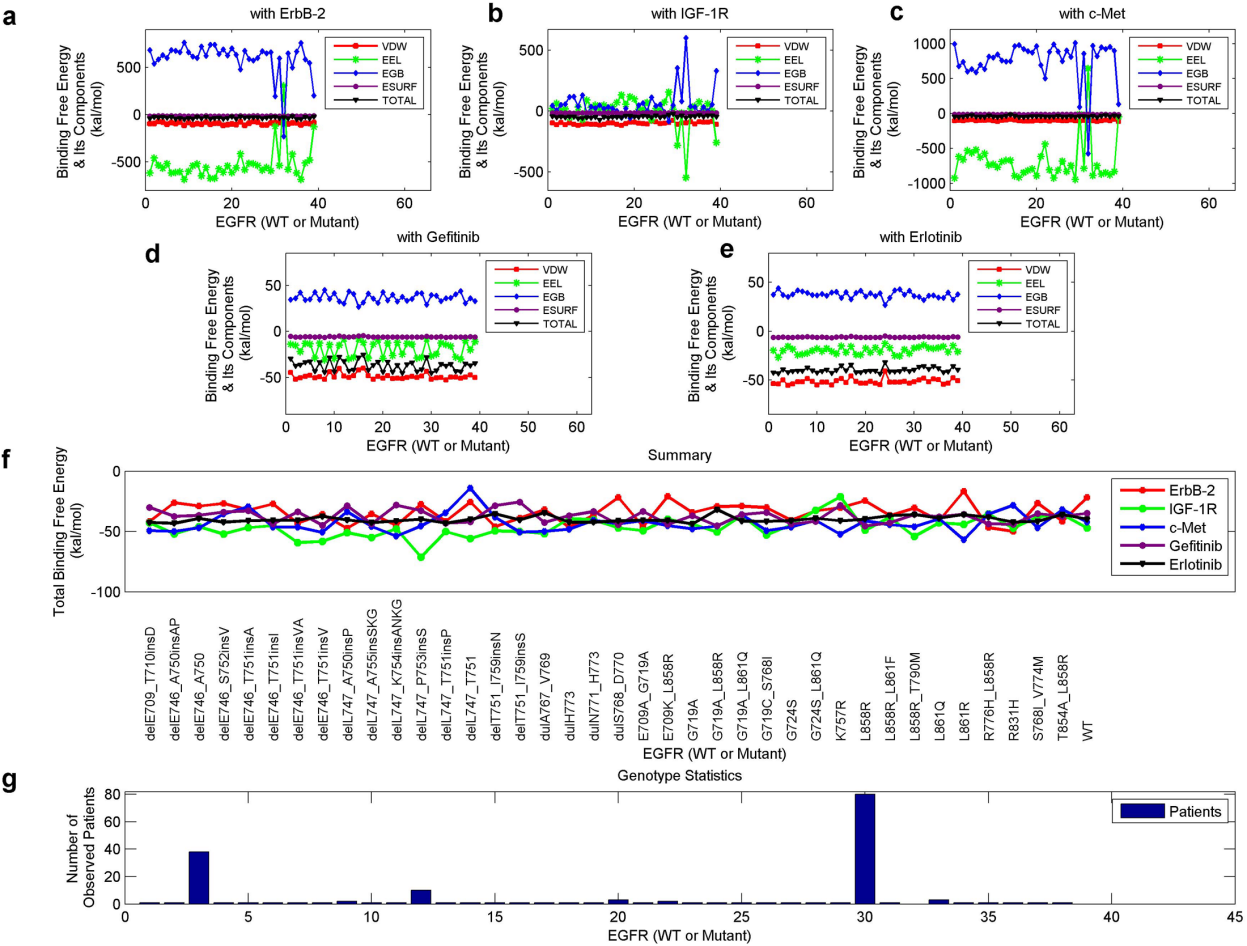
Binding free energy and its components of each mutant-inhibitor or mutant-partner system. (a) to (c), The energies for the systems that each involves a mutant and a dimerization partner (ErbB-2, IGF-1R or c-Met). (d) and (e), The energies for those systems concerning a mutant and an inhibitor (gefitinib or erlotinib). (f) The total binding free energies extracted from parts a to e, with the mutation types shown. (g) The statistical results of the mutation types among our NSCLC patients.

The distributions of binding free energies and their components, for each EGFR mutant (or WT protein) and an inhibitor or a dimerization partner, are presented in Fig 5a–5e. Furthermore, we comparably display the total binding free energies concerning the five kinds of systems in Fig 5f, with the mutation types listed. Also, the statistics of the 37 EGFR mutation types among our 168 patients are presented in Fig 5g, where the three peaks are delE746_A750, delL747_P753insS and L858R respectively. We can see that, type delL747_P753insS is somewhat distinct from the other two peaks whose energies concentrate more. In the following interpretations, we will analyze some specific mutants, including the well-known type L858R_T790M, for a further discussion.

**Fig 6:**
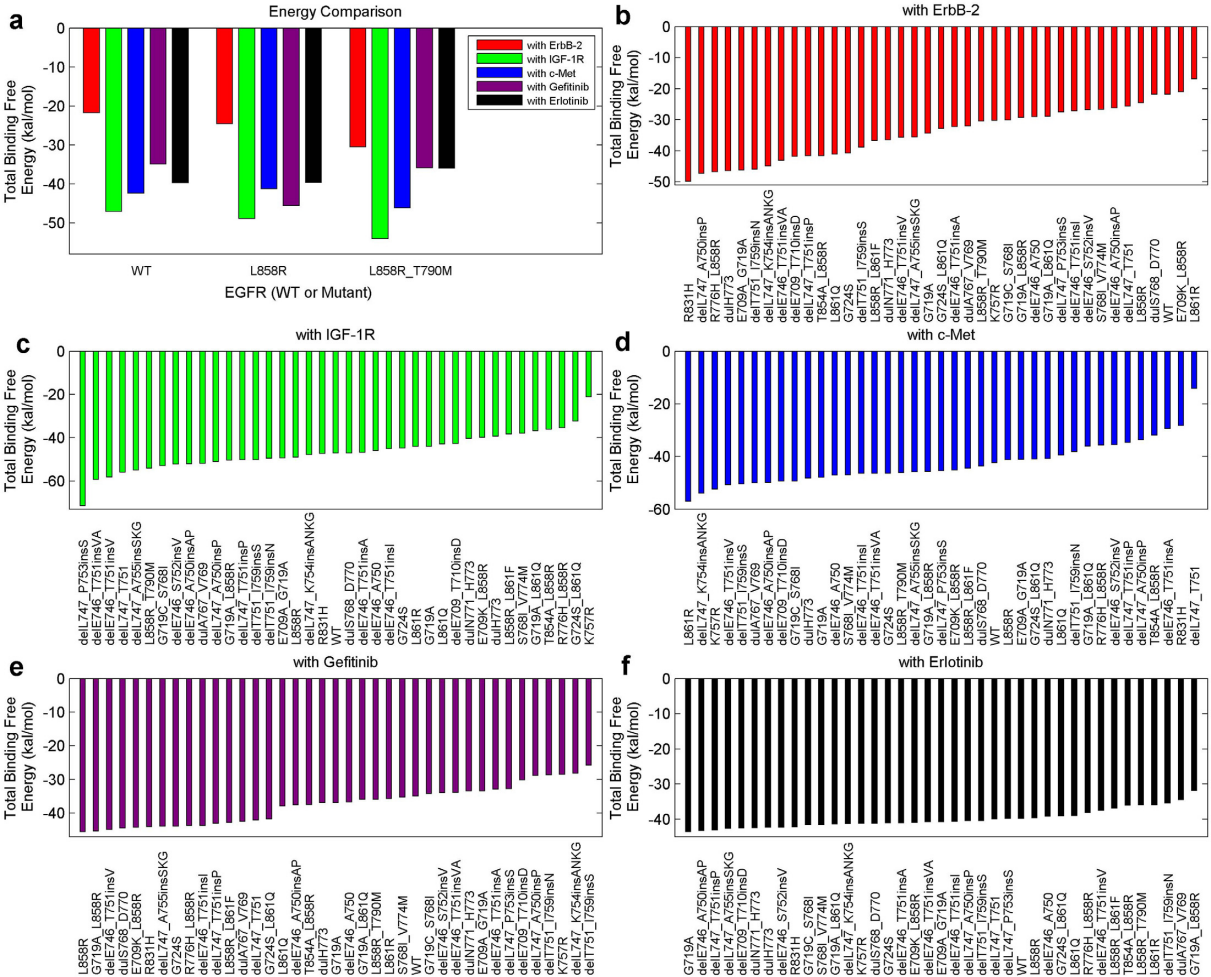
Total binding free energies of the mutant-inhibitor or mutant-partner systems, involving several specific mutants. (a), The total binding free energies between several important EGFR kinases (WT, L858R, L858R_T890M) and a dimerization partner (ErbB-2, IGF-1R, c-Met) or an inhibitor (gefitinib, erlotinib). (b) to (f), Ranked binding free energies for the systems regarding all of our mutants (37 types + WT + L858R_T790M) in an ascending order.

Now we analyze several specific instances by simply employing the total binding free energy. A low energy value, corresponding to a high absolute value, indicates a high binding affinity. Above all, we investigated the WT protein, mutant L858R (frequently occurs in NSCLC and responses well to TKIs) and mutant L858R_T790M (a double-point mutation that commonly triggers drug resistance). Fig 6a displays the binding free energies of these mutants with ErbB-2, IGF-1R, c-Met, gefitinib and erlotinib, respectively. Regarding the inhibitors, L858R binds more tightly with gefitinib than with the WT protein, while L858R_T790M binds less tightly with either gefitinib or erlotinib than with L858R. In addition, the binding of these mutants with ErbB-2 or IGF-1R gradually becomes tighter following the sequence of (i) the WT protein, (ii) L858R, and (iii) L858R_T790M. Regarding c-Met, L858R_T790M binds more tightly with it than with the WT protein or L858R. A tighter binding of the dimerization partners implies a stronger recovery of the downstream signaling pathways, thus brings negative effects to the efficacy of an inhibitor. The attenuated binding between mutant L858R_T790M and a TKI compared with that between L8585 and a TKI was well-verified in previous studies [19, 22, 23], sustaining our above analysis. As reported in [9, 34], strengthened EGFR-IGF-1R heterodimerization was found in NSCLC TKI-resistant cells than non-resistant cells, which is consistent to our results regarding mutants L858R (non-resistant) and L858R_T790M (TKI-resistant). Also, the increased EGFR-c-Met interaction in TKI-resistant cells (L858R_T790M) can be supported by the work in [47]. Combining these findings, we can explain the favorable efficacy of the inhibitors to L858R, and the resistance to L858R_T790M. Importantly, the mutant-partner interactions may be indispensable to the efficacy of an inhibitor or can cause the occurrence of drug resistance.

Subsequently, we sorted the total binding free energies concerning all the mutation types of our patients in Fig 6b–6f, with the mutation types listed. As an example, L858R has a largest binding free energy with gefitinib, a low binding free energy with ErbB-2 (fifth from the last), and an average binding affinity with either IGF-1R, c-Met or erlotinib.

**Fig 7:**
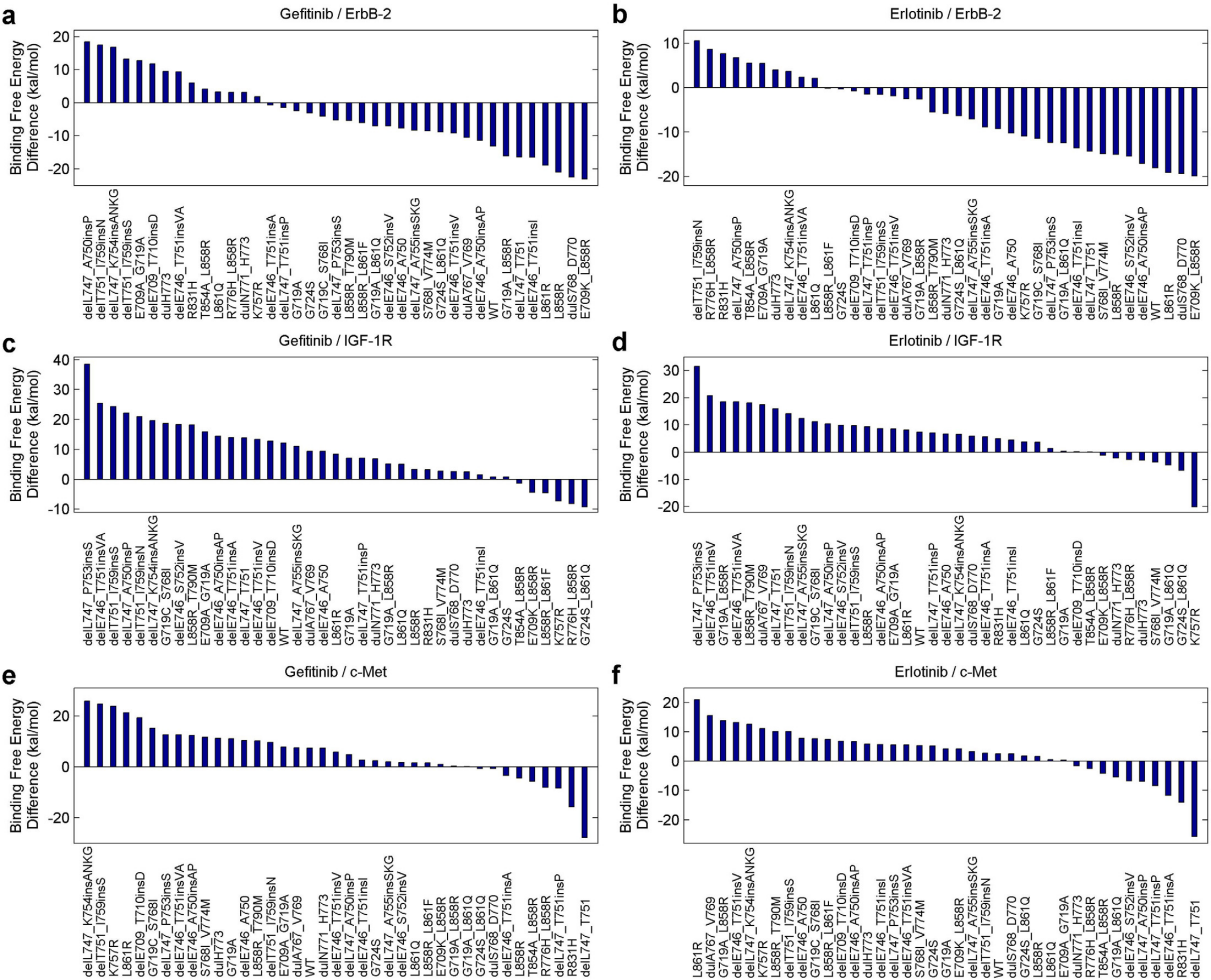
The difference between the total binding free energies of the mutant-inhibitor system and the mutant-partner system for each mutant. (a) and (b), The binding free energy differences between the systems involving ErbB-2 and the two inhibitors (gefitinib and erlotinib), with the values ranked in the descending order. (c) and (d), The similar binding free energy differences between the systems concerning IGF-1R and the two inhibitors. (e) and (f), The binding free energy differences between the systems concerning c-Met and the two inhibitors.

Since the mutant-partner interactions can bring a negative influence to the efficacy of an inhibitor while the mutant-inhibitor interactions produce a positive one, we further extract the total binding free energy difference between them for a detailed analysis. Fig 7a shows the energy difference of the mutant-gefitinib and the mutant-ErbB-2 systems for each mutant, with the differences ranked in a descending order. Similarly, Fig 7b displays the energy difference between the mutant-erlotinib and mutant-ErbB-2 systems for each mutant. Each total binding free energy value has a negative sign; therefore, a larger difference value indicates a lower efficacy of the inhibitor or a higher drug resistance level. Likewise, Fig 7c–7f reveal how the energy differences, concerning IGF-1R/c-Met and the two inhibitors, distribute.

Fig 7 uncovers that, L858R corresponds to a largely-negative value in either part a, b, or e, and an averaged-positive value in part c, d, or f. Besides, in all these six scenarios, L858R_T790M ranks higher than L858R, implying a higher drug resistance level. delL747_P753 is a frequent-occurred mutation associated with deletions in exon 19 [72, 73], and it was commonly reported to have poor or even worst clinical outcomes among NSCLC patients [16, 53, 73]. As shwon in [53], delL747_P753insS has the shortest PFS among all the examined mutations, indicating a high drug resistance level. Furthermore, in [16], the mutation was concluded to trigger increased growth factor signaling and confer susceptibility to inhibitors. Strikingly, delL747_P753insS commonly occurs among our patients, and ranks the first in Fig 7c and 7d, possessing a larger difference than other mutations. Also, it ranks higher in either part e or f. It implies that, IGF-1R or c-Met may be a crucial factor that influences the EGFR signaling and promotes the drug resistance level in NSCLC treatments. In the subsequent section, we will bring the energy components into the discussion, to broaden our investigation.

### Regression Results from Binding Free Energy Components to PFS

**Fig 8:**
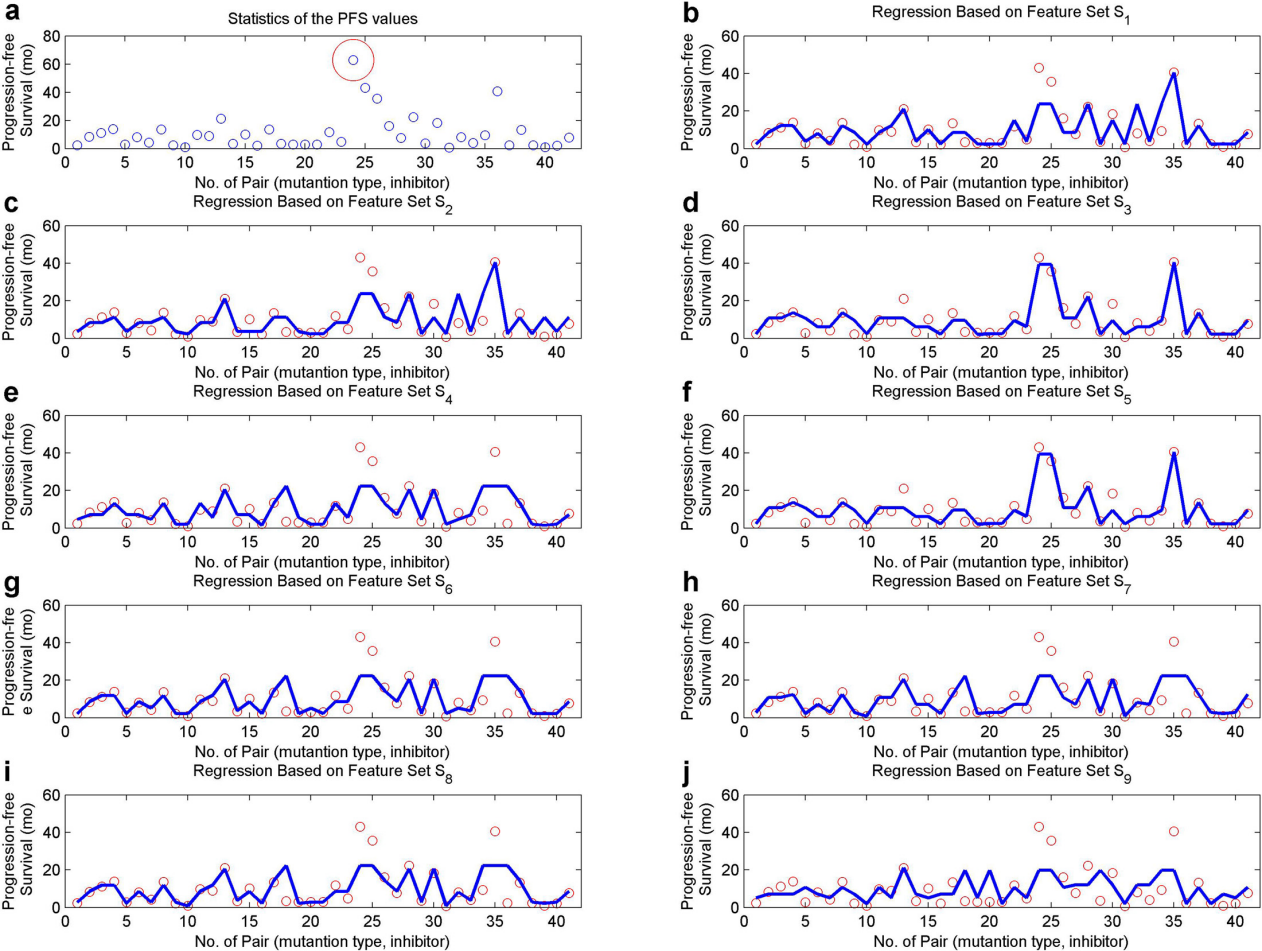
A regression analysis on different feature sets and the median PFS values for patient groups. (a) The median PFS values (in months) for all the patient groups, with an outlier circled. Each blue point represents a patient group, where the included patients share the same EGFR mutation type and inhibitor. (b) to (j), The regression results based on features sets *S*_1_ to *S*_9_, after an outlier-removal from part a. The blue curves indicate the predicted PFS values for our patient groups.

In order to explore the effects of an inhibitor to a specific mutation type, we categorized our patients into groups where patients in each group share the same mutation type and inhibitor. Each group corresponds to a PFS extracted from the PFSs of the component patients, either as a median or an evolved average (the largest and lowest values were excluded). The medians scatter in Fig 8a, where the red circle is an apparent outlier and was discarded in our studies.

We regarded the previously-obtained energies as features for each group, and aimed to construct a mapping from the energy to the PFS. The regression trees [65], was applied in the study. To ease our succeeding illustrations, we notate the binding free energies or their components in different scenarios as follows,

- *MI*_1_, *MI*_2_, *MI*_3_ and *MI*_4_ represent the four components (VDW, EEL, EGB and ESURF) of the binding free energy between the mutant and inhibitor concerning each patient group, and *MI* indicates the total binding free energy.
- *ME*_1_, *ME*_2_, *ME*_3_ and *ME*_4_ are the components of the binding free energy between a mutant and its partner ErbB-2, and ME is the total binding free energy.
- *MG*_1_, *MG*_2_, *MG*_3_ and *MG*_4_ stand for the components of the binding free energy regarding a mutant and its partner IGF-1R, and MG is the total binding free energy.
- *MC*_1_, *MC*_2_, *MC*_3_ and *MC*_4_ stand for the components of the binding free energy regarding a mutant and its partner c-Met, and MC is the total binding free energy.

To compare the influences of different factors to the PFS of an inhibitor, which implies the resistance level to this inhibitor, we constructed feature sets *S*_1_ to *S*_9_ as defined below,

- *S*_1_ = (*MI*_1_, *MI*_2_, *MI*_3_, *MI*_4_),
- *S*_2_ = (*MI*_1_, *MI*_2_, *MI*_3_, *MI*_4_, *ME*_1_, *ME*_2_, *ME*_3_, *ME*_4_),
- *S*_3_ = (*MI*_1_, *MI*_2_, *MI*_3_, *MI*_4_, *MG*_1_, *MG*_2_, *MG*_3_, *MG*_4_),
- *S*_4_ = (*MI*_1_, *MI*_2_, *MI*_3_, *MI*_4_, *MC*_1_, *MC*_2_, *MC*_3_, *MC*_4_),
- *S*_5_ = (*MI*_1_, *MI*_2_, *MI*_3_, *MI*_4_, *ME*_1_, *ME*_2_, *ME*_3_, *ME*_4_, *MG*_1_, *MG*_2_, *MG*_3_, *MG*_4_),
- *S*_6_ = (*MI*_1_, *MI*_2_, *MI*_3_, *MI*_4_, *ME*_1_, *ME*_2_, *ME*_3_, *ME*_4_, *MC*_1_, *MC*_2_, *MC*_3_, *MC*_4_),
- *S*_7_ = (*MI*_1_, *MI*_2_, *MI*_3_, *MI*_4_, *MC*_1_, *MC*_2_, *MC*_3_, *MC*_4_, *MG*_1_, *MG*_2_, *MG*_3_, *MG*_4_),
- *S*_8_ = (*MI*_1_, *MI*_2_, *MI*_3_, *MI*_4_, *ME*_1_, *ME*_2_, *ME*_3_, *ME*_4_, *MG*_1_, *MG*_2_, *MG*_3_, *MG*_4_, *MC*_1_, *MC*_2_, *MC*_3_, *MC*_4_),
- *S*_9_ = (*MI*, *ME*, *MG*, *MC*).

A regression analysis was conducted on each feature set, with the results exhibited in Fig 8b–8j. Besides, the relative absolute errors (RAEs) and root relative square errors (RRSEs), generally used for evaluating the regression performances, are listed in Table 1 for these scenarios in both cases involving the median PFSs and average PFSs. As revealed in Fig 8, the interaction in a mutant-inhibitor system (feature set *S*_1_) is a fundamental element for predicting the drug resistance level concerning gefitinib or erlotinib. Bringing in the interaction in a mutant-ErbB-2 or mutant-c-Met system (feature set *S*_2_ or *S*_4_) leads to a higher RAE (0.4796, 0.4854 / 0.5694, 0.5439) or RRSE (0.5742, 0.5694 / 0.6946, 0.6937) in both scenarios. It may reveal that, ErbB-2 is not a significant factor in controlling the drug resistance in NSCLC treatments, while it has been broadly acknowledged to be important in the treatments of breast cancer [33, 74]. c-Met was well-verified to be an important co-factor for ErbB-3 to activate ErbB-3 signaling, in lung cancer cells developing resistance to TKIs [46, 47]. Therefore, in this scenario, the major role of c-Met in communicating with ErbB family receptors may be decoded by c-Met-ErbB-3 interactions. These will be discussed in the following section. It is noteworthy that, the adoption of the interaction in a mutant-IGF-1R system (feature set *S*_3_) can improve the regression performances with a lower RAE (0.3168 / 0.3239) or RRSE (0.3437 / 0.3433), in both situations. IGF-1R may behave as a vital role in regulating the EGFR signaling pathways, therefore influences the resistance level to an inhibitor in NSCLC treatment. Feature set *S*_9_ corresponds to the worst performance, indicating the concealed information in the components of binding free energies.

**Table 1:** RAE and RRSE values for the regressions.

**Median PFS**									
**Set**	***S***_***1***_	***S***_***2***_	***S***_***3***_	***S***_***4***_	***S***_***5***_	***S***_***6***_	***S***_***7***_	***S***_***8***_	***S***_***9***_
**Measure**									
**RAE**	0.3434	0.4796	**0.3168**	0.5694	**0.3168**	0.5206	0.5346	0.5061	0.6980
**RRSE**	0.5125	0.5742	**0.3437**	0.6946	**0.3437**	0.6836	0.6893	0.6824	0.7760
**Evolved Average PFS**									
**Set**	***S***_***1***_	***S***_***2***_	***S***_***3***_	***S***_***4***_	***S***_***5***_	***S***_***6***_	***S***_***7***_	***S***_***8***_	***S***_***9***_
**Measure**									
**RAE**	0.4021	0.4854	**0.3239**	0.5439	**0.3239**	0.4996	0.5288	0.4775	0.8091
**RRSE**	0.5299	0.5694	**0.3433**	0.6937	**0.3433**	0.6832	0.6935	0.6802	0.8160

Different feature sets *S*_1_ to *S*_9_ were tested in the regression analysis, with the median PFS or evolved average PFS applied. *S*_1_ indicates the mutant-inhibitor interaction; *S*_2_ further brings in the mutant-ErbB-2 interaction; *S*_3_ focuses on both the mutant-IGF-1R and mutant-inhibitor interactions; and *S*_4_ includes both the mutant-c-Met and mutant-inhibitor interactions. *S*_5_ to *S*_7_ each includes the mutant-inhibitor interaction and two kinds of mutant-partner interactions, and *S*_8_ or *S*_9_ considers all the four kinds of interactions. Specifically, *S*_1_ to *S*_8_ apply the binding free energy components, while *S*_9_ only adopts the total binding free energy.

**Fig 9:**
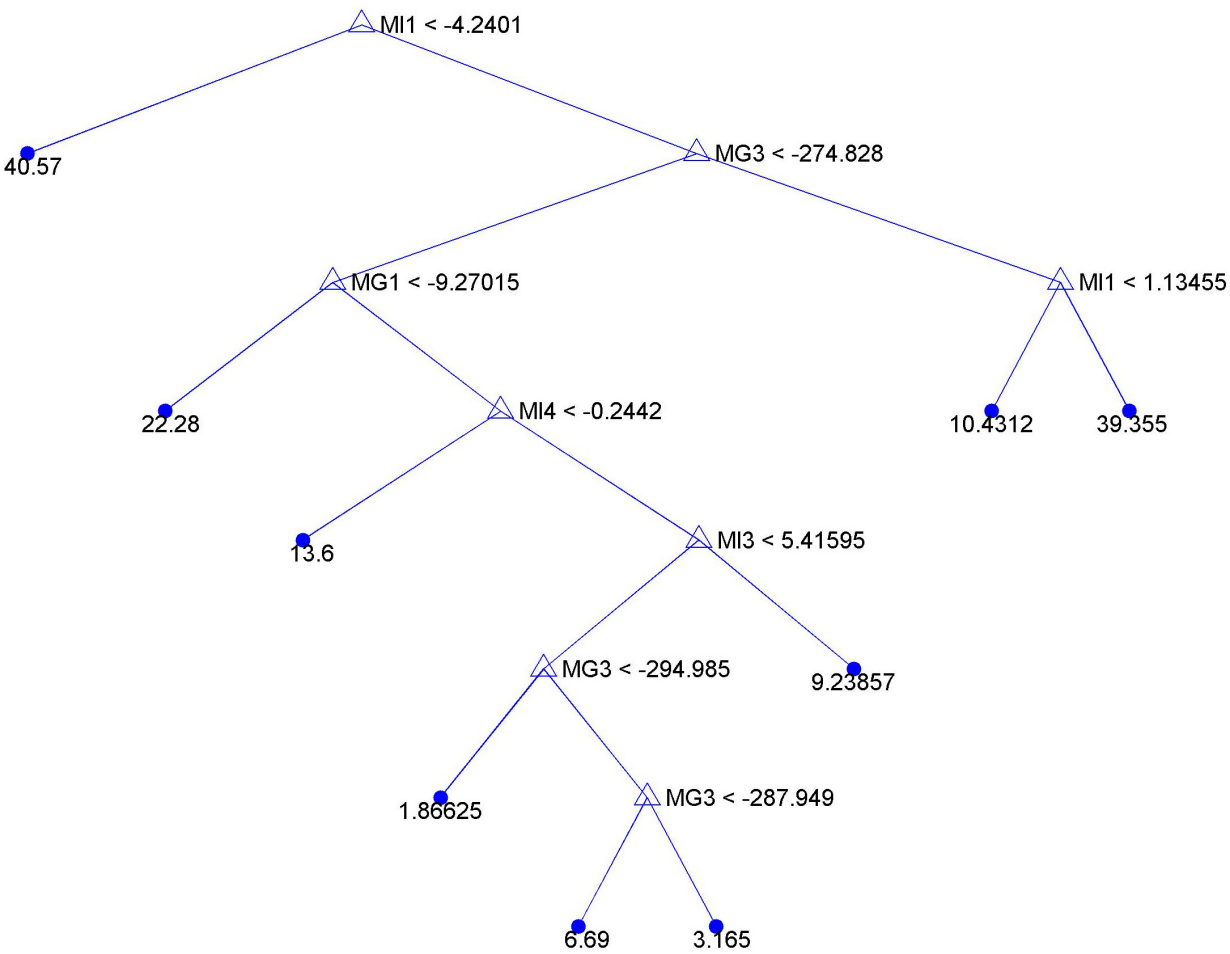
A regression tree generated based on feature set *S*_3_. *S*_3_ = (*MI*_1_, *MI*_2_, *MI*_3_, *MI*_4_, *MG*_1_, *MG*_2_, *MG*_3_, *MG*_4_), which includes the interaction patterns, characterized by binding free energy components, in a mutant-IGF-1R or mutant-inhibitor system for each patient group. Patients in the same group share the same EGFR mutation type and inhibitor.

Ultimately, an example regression tree based on feature set *S*_3_ is presented in Fig 9. As shown in this figure, components VDW (*MG*_1_) and EGB (*MG*_3_) of the binding free energies, in mutant-inhibitor systems, participate in the tree construction. Especially, *MG*_3_ occurs three times, implying this component may be an important factor to influence TKI efficacy in NSCLC treatments. Moreover, components VDW (*MI*_1_), EGB (*MI*_3_), and ESURF (*MI*_4_) of the binding free energies, in mutant—IGF-1R systems, primarily contribute to the tree construction. The VDW component is adopted twice for this prediction, indicating it may be useful in the drug resistance prediction in lung cancer studies. This tree model can be further improved, when involving more accurate structural analysis and sufficient patient data in future studies.

### Investigation of ErbB-3-partner Interactions Based on Binding Free Energies

**Fig 10:**
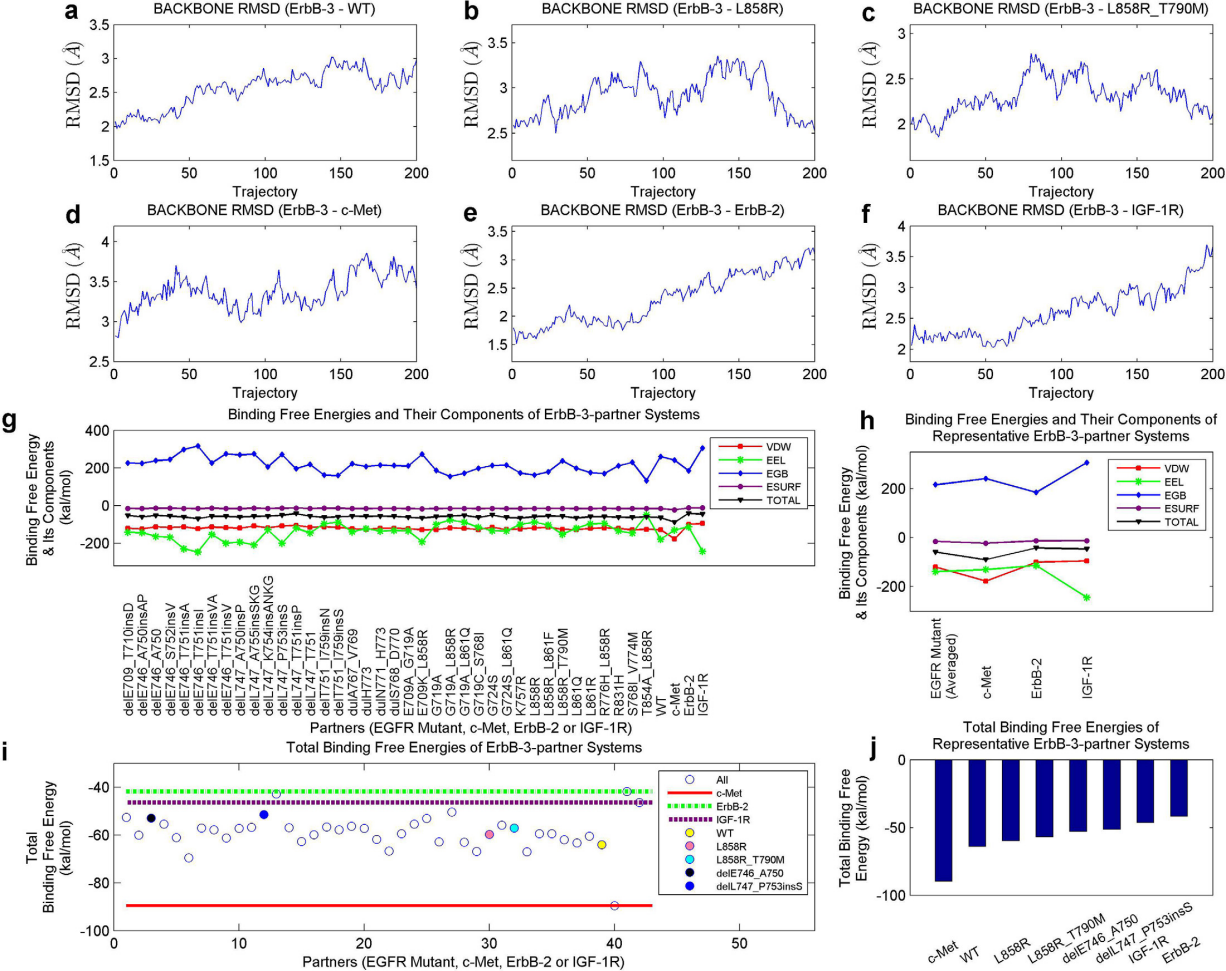
Computational results for ErbB-3-partner systems. (a) to (f), Respective backbone RMSD curves of ErbB-3—WT, ErbB-3—L858R, ErbB-3—L858R_T790M, ErbB-3—c-Met, ErbB-3—ErbB-2 and ErbB-3—IGF-1R systems, in the 2-ns production MD simulations. (g) Binding free energies and their components of ErbB-3-partner systems. (h) Binding free energies and their components of ErbB-3—EGFR mutant (averaged), ErbB-3—c-Met, ErbB-3—ErbB-2 and ErbB-3—IGF-1R systems. (i) Total binding free energy comparison among ErbB-3-partner systems. Binding free energies of ErbB-3—c-Met, ErbB-3—ErbB-2 and ErbB-3—IGF-1R systems are labeled with lines, and those of systems involving WT EGFR, L858R, L858R_T790, delE746_A750 and delL747_P753insS are marked with solid spheres. (j) Ranked total binding free energies of representative ErbB-3-partner systems.

Binding free energies of ErbB-3-partner systems, involving ErbB-3 and its partners EGFR mutants, c-Met, ErbB-3 and IGF-1R, were similarly derived as those of mutant-partner systems. The stabilizations of these systems, in the 2-ns production MD simulation, were validated and the RMSD curves of several instances are shown in Fig 10a–10f. Binding free energies and their components for the examined systems are shown in Fig 10g. In addition, the results involving EGFR mutants were averaged, and compared to ErbB-3—c-Met, ErbB-3—ErbB-2 and ErbB-3—IGF-1R systems (Fig 10h).

Further, we investigated the total binding free energies of ErbB-3-partner systems (Fig 10i), and ranked several representatives (Fig 10j). In Fig 10i and 10j, both ErbB-2 and IGF-1R bind less tighter with ErbB-3 than majority of EGFR proteins, while c-Met presents a significantly-strong binding with ErbB-3 compared to these EGFR mutants. This may imply the positive contribution of c-Met in promoting the ErbB-3 signaling in lung cancer cells [46, 47]. Besides, mutants such as L858R, L858R_T790M, delE746_A750 and delL747_P753insS have a lower binding affinity with ErbB-3 than the WT protein does, suggesting larger differences between such interactions and the ErbB-3—c-Met interaction. As mutations L858R_T790M and delL747_P753insS largely corresponded to poor clinical outcomes and active TKI resistance [16, 22, 23, 53], their decreased binding with ErbB-3 may encourage the binding between c-Met and ErbB-3, which strengthens the ErbB-3 downstream signaling. After considering the energy differences between each ErbB-3-mutant system and the ErbB-3-c-Met system, the above-mentioned regression performances can be further improved to 0.3036/0.3359 and 0.2965/0.3338 for RAE/RRSE in the two secnarios (Median PFS and Evolved Average PFS).

### Summary and Future Works

Computational biology has become an indispensable branch of life sciences [49–52]. The computational and structural studies nourished many fields, such as the prediction of molecular structures or interactions [75], the construction of the protein function network [76], and the discovery of innovative drugs [77]. In our work, 37 EGFR mutants, extracted from our 168 NSCLC patients, were investigated based on their 3D structures. Both the mutant-inhibitor and mutant-partner interactions were characterized using the binding free energy and its components, based on the molecular dynamics of these systems. Specifically, mutant L858R_T790M has a weaker connection with the inhibitors (gefitinib and erlotinib) but a stronger interaction with the dimerization partners (ErbB-2, IGF-1R and c-Met), compared to mutant L858R. Further, mutant delL747_P753insS corresponds to an apparently high difference between its interactions with IGF-1R and an inhibitor (gefitinib or erlotinib). These observations may support the significant contribution of IGF-1R to the resistance level concerning an inhibitor. Moreover, the binding free energy components were brought in to construct a generalized regression model for predicting the PFS of an inhibitor. Regression trees [65], involving different energy feature sets, confirmed the positive influence of IGF-1R in regulating the drug resistance level in NSCLC treatments. As a supplementary study, interactions between ErbB-3 and its potential partners were analyzed and compared, similarly relying on the binding free energies. c-Met corresponded to a stronger binding with ErbB-3 than EGFR mutants, suggesting its important influence in strengthening ErbB-3 signaling.

The study or prediction of drug resistance level in a cancer therapy is vital for cancer-related research. To explore the links (proteins or complexes) in cancer signaling networks can bring promising results. In our work, the contribution of EGFR and ErbB-3 heterodimerization to the drug resistance mechanism in NSCLC treatments was analyzed, according to a structural perspective. The significant influences of IGF-1R and c-Met in the EGFR/ErbB-3 downstream signaling were further verified. Dually-targeting inhibitors, such as those targeting both EGFR and IGF-1R, may produce a good efficacy. These findings can encourage the development of innovative drug discovery or specialized therapy design. In future studies, proteins relating to different links in cancer signaling pathways can be our targets, based on which we will gain a deeper insight into the drug resistance mechanism and further improve our prediction model. As another part of our future goals, more efficient strategies should be explored to further decrease the computational costs of calculating the binding affinity of a protein-inhibitor or protein-protein system.

## Supporting Information

S1 File Binding free energy tables of EGFR-inhibitor, EGFR-partner, and ErbB-3-partner systems. Binding free energies and their components of EGFR mutant—Gefitinib systems **(Table A)**. Binding free energies and their components of EGFR mutant—Erlotinib systems **(Table B)**. Binding free energies and their components of EGFR mutant—ErbB-2 systems **(Table C)**. Binding free energies and their components of EGFR mutant—IGF-1R systems **(Table D)**. Binding free energies and their components of EGFR mutant—c-Met systems **(Table E)**. Binding free energies and their components of ErbB-3-partner systems **(Table F)**.
(DOC)
