# Supplementary material for: Contribution of EGFR and ErbB-3 Heterodimerization to the EGFR Mutation-Induced Gefitinib- and Erlotinib-Resistance in Non-Small-Cell Lung Carcinoma Treatments
Source: PLoS One. 2015 May 20;10(5):e0128360. doi: 10.1371/journal.pone.0128360 (PMC4439022; doi:10.1371/journal.pone.0128360)
Supplement: S1 File — Binding free energies and their components of EGFR mutant—Gefitinib systems (Table A). Binding free energies and their components of EGFR mutant—Erlotinib systems (Table B). Binding free energies and their components of EGFR mutant—ErbB-2 systems (Table C). Binding free energies and their components of EGFR mutant—IGF-1R systems (Table D). Binding free energies and their components of EGFR mutant—c-Met systems (Table E). Binding free energies and their components of ErbB-3-partner systems (Table F). (DOC) [file pone.0128360.s001.doc]

Contribution of EGFR and ErbB-3 Heterodimerization to the EGFR Mutation-induced Gefitinib- and Erlotinib-resistance in Non-Small-Cell Lung Carcinoma Treatments

Debby D. Wang^1^*, Lichun Ma^1^, Maria P. Wong^2^, Victor H.F. Lee^2^, Hong Yan^1^

^1^*Department of Electronic Engineering, City University of Hong Kong, Kowloon, Hong Kong*.

^2^*Li Ka Shing Faculty of Medicine, The University of Hong Kong, Pokfulam, Hong Kong*.

***Corresponding Author:** Debby Dan Wang

Mailing Address: 2384 Fong Yun-Wah Building, City University of Hong Kong, Kowloon, Hong Kong

Phone Number: +852-96462175

E-mail Address: danwang6-c@my.cityu.edu.hk

**S1 File. Binding free energy tables of EGFR-inhibitor, EGFR-partner, and ErbB-3-partner systems.** Binding free energies and their components of EGFR mutant - Gefitinib systems **(Table A).** Binding free energies and their components of EGFR mutant - Erlotinib systems **(Table B).** Binding free energies and their components of EGFR mutant - ErbB-2 systems **(Table C).** Binding free energies and their components of EGFR mutant - IGF-1R systems **(Table D).** Binding free energies and their components of EGFR mutant - c-Met systems **(Table E).** Binding free energies and their components of ErbB-3-partner systems **(Table F).**

**Table A. Binding free energies and their components of EGFR mutant - Gefitinib systems.** This table lists the binding free energies and their components for EGFR mutants (including the WT protein) with an inhibitor (gefitinib). These components encompass energy components of (A) van der Waals forces (VDW), (B) electrostatic interactions (EEL), and (C) the electrostatic (EGB) and (D) non-electrostatic (ESURF) contributions of the solvation free energies.

**Table B. Binding free energies and their components of EGFR mutant - Erlotinib systems.** This table lists the binding free energies and their components for EGFR mutants (including the WT protein) with an inhibitor (erlotinib). These components encompass energy components of (A) van der Waals forces (VDW), (B) electrostatic interactions (EEL), and (C) the electrostatic (EGB) and (D) non-electrostatic (ESURF) contributions of the solvation free energies.

**Table C. Binding free energies and their components of EGFR mutant - ErbB-2 systems.** This table lists the binding free energies and their components for EGFR mutants (including the WT protein) with a dimerization partner (ErbB-2). These components encompass energy components of (A) van der Waals forces (VDW), (B) electrostatic interactions (EEL), and (C) the electrostatic (EGB) and (D) non-electrostatic (ESURF) contributions of the solvation free energies.

**Table D. Binding free energies and their components of EGFR mutant – IGF-1R systems.** This table lists the binding free energies and their components for EGFR mutants (including the WT protein) with a dimerization partner (IGF-1R). These components encompass energy components of (A) van der Waals forces (VDW), (B) electrostatic interactions (EEL), and (C) the electrostatic (EGB) and (D) non-electrostatic (ESURF) contributions of the solvation free energies.

**Table E. Binding free energies and their components of EGFR mutant - c-Met systems.** This table lists the binding free energies and their components for EGFR mutants (including the WT protein) with a dimerization partner (c-Met). These components encompass energy components of (A) van der Waals forces (VDW), (B) electrostatic interactions (EEL), and (C) the electrostatic (EGB) and (D) non-electrostatic (ESURF) contributions of the solvation free energies.

**Table F. Binding free energies and their components of ErbB-3-partner systems.** This table lists the binding free energies and their components for ErbB-3 with a dimerization partner (EGFR mutants, cMet, ErbB-2 and IGF-1R). These components encompass energy components of (A) van der Waals forces (VDW), (B) electrostatic interactions (EEL), and (C) the electrostatic (EGB) and (D) non-electrostatic (ESURF) contributions of the solvation free energies.
